# Selection criteria for high-yielding and early-flowering bread wheat hybrids under heat stress

**DOI:** 10.1371/journal.pone.0236351

**Published:** 2020-08-12

**Authors:** Ibrahim Al-Ashkar, Majed Alotaibi, Yahya Refay, Abdelhalim Ghazy, Adel Zakri, Abdullah Al-Doss

**Affiliations:** 1 Plant Production, College of Food and Agriculture Sciences, King Saud University, Riyadh, Saudi Arabia; 2 Agronomy Department, Faculty of Agriculture, Al-Azhar University, Cairo, Egypt; North Dakota State University, UNITED STATES

## Abstract

Hybrid performance during wheat breeding can be improved by analyzing genetic distance (GD) among wheat genotypes and determining its correlation with heterosis. This study evaluated the GD between 16 wheat genotypes by using 60 simple sequence repeat (SSR) markers to classify them according to their relationships and select those with greater genetic diversity, evaluate the correlation of the SSR marker distance with heterotic performance and specific combining ability (SCA) for heat stress tolerance, and identify traits that most influence grain yield (GY). Eight parental genotypes with greater genetic diversity and their 28 F_1_ hybrids generated using diallel crossing were evaluated for 12 measured traits in two seasons. The GD varied from 0.235 to 0.911 across the 16 genotypes. Cluster analysis based on the GD estimated using SSRs classified the genotypes into three major groups and six sub-groups, almost consistent with the results of principal coordinate analysis. The combined data indicated that five hybrids showed 20% greater yield than mid-parent or better-parent. Two hybrids (P2 × P4) and (P2 × P5), which showed the highest performance of days to heading (DH), grain filling duration (GFD), and GY, and had large genetic diversity among themselves (0.883 and 0.911, respectively), were deemed as promising heat-tolerant hybrids. They showed the best mid-parent heterosis and better-parent heterosis (BPH) for DH (-11.57 and -7.65%; -13.39 and -8.36%, respectively), GFD (12.74 and 12.17%; 12.09 and 10.59%, respectively), and GY (36.04 and 20.04%; 44.06 and 37.73%, respectively). Correlation between GD and each of BPH and SCA effects based on SSR markers was significantly positive for GFD, hundred kernel weight, number of kernels per spike, harvest index, GY, and grain filling rate and was significantly negative for DH. These correlations indicate that the performance of wheat hybrids with high GY and earliness could be predicted by determining the GD of the parents by using SSR markers. Multivariate analysis (stepwise regression and path coefficient) suggested that GFD, hundred kernel weight, days to maturity, and number of kernels per spike had the highest influence on GY.

## Introduction

Wheat (*Triticum aestivum* L.), one of the most prominent food crops worldwide, is one of the most essential sources of protein in humans. It represents 17% of the global crop area, feeding about 40% of world’s population and providing 20% of the total diet calories [1]. In addition, wheat straw is an important component of animal feed. Agricultural productivity is remarkably affected by extreme weather events. Multiple challenges such as high temperature stress and reduced water availability are the major concerns for all countries in the Arab region [2]. In addition to these, the incidence of diseases and pest infestation is increasing with global warming [3]. All these negative influences threaten the sustainability of grain crop production. Decreased wheat productivity has caused devastating economic and sociological impacts [4]. In particular, the steady rise in population, loss of agricultural lands to sustainable urbanization, and decrease in resource availability owing to climate change pose serious threats to the safe production of wheat [3].

Wheat production needs to be continually increased by 2% each year to meet the basic needs of the increasing human population. The difference between wheat production and consumption has been bridged by targeting breeding efforts toward increasing wheat productivity by using high-yielding and early-maturing cultivars for use in intensive cropping systems and to avoid hot winds at the end of the agricultural season during grain filling [5]. Heat intensity remarkably influences flowering, pollination, and grain filling and is a serious challenge to sustain high production. Continual and terminal high temperature stresses are the two major impediments to wheat production [5]. The impact of extreme heat waves has been analyzed in wheat [6, 7]. An increase of 3–4°C of seasonal temperature has been shown to decrease wheat yield by 15–35% in Africa and Asia and by 25–35% in the Middle East during the grain-filling period [8, 9]. Thus, developing new high-yielding genotypes that are early maturing and have extended grain filling duration is necessary. Evaluation of the genetic parameters for agronomic and physiological characteristics is important to determine the best parents and hybrids that can be used in breeding programs for selecting promising lines/varieties of wheat tolerant to biotic and abiotic stresses.

During wheat development, selection of parents for crossing requires careful characterization, germplasm assessment, and crop variety identification. Most recently, molecular DNA markers have been occasionally used for this process [10]. Biotechnology has the potential to facilitate and promote sustainable agriculture and rural development, especially because it can provide renewable and sustainable genetic inputs. Molecular marker technologies have been used in genetic diversity studies, molecular-assisted selection (MAS), paternity analysis, quantitative trait loci mapping (QTL), cultivar identification, phylogenetic relationship analysis, and genetic mapping. DNA fingerprinting markers play a major role in revealing polymorphisms. The selection of accessions is more accurate when genetic markers rather than phenotypic traits, which are robustly influenced by environmental factors and thus cannot be assessed accurately, are used to produce a highly specific pattern of bands for each individual. Molecular markers can also be efficiently used for variation and phylogenetic analyses. Simple sequence repeat (SSR) markers, also known as microsatellites, are among the most important molecular markers; they are multi-allele, co-dominant, highly informative, relatively highly abundant, widely distributed across the genome, and reproducible [11, 12]. SSRs are very advantageous for various applications in genetic research and breeding, population structure analysis, gene mapping, assisted selection for crop improvement, and genetic diversity estimation of wheat cultivars and lines [13–18].

Hybrids are widely used for maize and rice. Exploitation of hybrid vigor at the commercial level by developing hybrid wheat is considered as one of the promising approaches for increasing wheat productivity; however, developing a viable hybrid system for bread wheat is challenging owing to the large and hexaploid wheat genome [19]. Wheat is monoecious; therefore, a line designated as a female parent should not be allowed to produce pollen capable of fertilization. Hand emasculation, cytoplasmic male sterility (CMS), and chemical hybridization agents (CHAs) are used to obtain male sterility in plants. However, mechanical emasculation is laborious, very expensive, and time consuming [10]; hence, it is performed in research studies only. Wilson and Ross [20] first used CMS to develop a wheat hybrid; this was confirmed by He et al. [21]. However, genetic progress in developing agronomically improved R-lines by using CMS remains the most significant obstacle in the continued development of hybrid wheat. CHAs are associated with problems of toxicity and selectivity. Hence, their commercialization is difficult owing to their limited application under the prevailing environmental conditions [22]. Thus, developing a series of technological advances for hybrid wheat programs is essential. For this, major changes in floral development and architecture are required to separate the sexes and force outcrossing. Male sterility is the best method to block self-fertilization, and flower structure modification might enhance pollen access. This represents a key step toward developing a robust hybridization platform in wheat [19, 22]. Hybrid seed production costs can be reduced by using a reliable and an inexpensive system that forces outcrossing.

Population structure can be predicted by determining the genetic identity, and the breeding values of ungenotyped descendants can be inferred by conducting pedigree-based simulations [23, 24]; the selection for specific traits can assist in the selection of crossing parents to combine diverse germplasm in a breeding program. Crossing parents can be selected on the basis of their genetic distance (GD), in order to maximize the overall genetic diversity and potential for genetic gain in the progeny [25]. Crosses between genetically distant parents may present a wider genetic variance available for selection [26] as well as result in greater potential for heterosis and higher performance of F_1_ hybrid varieties [27–29]. Heterosis is caused by allelic and non-allelic interactions of genes in either homozygotes or heterozygotes under the influence of a particular environment. Although heterosis has been observed in wheat, its level is widely different among F_1_ crosses. Therefore, in wheat, the commercial use of hybrids is restricted to cases in which heterosis is sufficient to cover the requirements of the extra cost incurred to produce hybrid wheat seeds. Therefore, further studies are warranted to identify the cross combinations that express desirable heterosis compared with their parents [3, 30–32]. Knowledge of heritability of a trait guides plant breeders to predict the behavior of succeeding generations and the response to selection. Wheat breeders have exploited “transgressive segregation” in hybridization programs by selecting superior traits that increase yield in target environments [33].

Knowledge of the relative importance of additive and non-additive gene action is necessary for a plant breeder to develop an effective hybridization program. The term combining ability refers to the capacity or ability of a genotype to transmit its outstanding performance to the progeny. The value of a genotype depends on its ability to produce superior hybrids in combination with other genotypes [34–36]. This study aimed to estimate the genetic variability of 16 genotypes according to their relationships and select more genetically diverse hybrids by using SSRs, characterize the population structure of elite wheat germplasm selected, and access superior hybrids from better parents to cover their production costs. Furthermore, this study intended to obtain hybrids that are early maturing and high yielding and have extended grain filling duration, estimate the correlations of SSR marker distance with heterosis and SCA, and identify traits that most influence grain yield.

## Materials and methods

### Experimental material

In this study, a set of 16 genotypes of bread wheat was selected from different ecological regions on the basis of the presence of wide genetic differences between them with respect to certain agronomic traits (S1 Table). This included eight varieties from the Agricultural Research Center, Egypt. The remaining eight doubled haploid lines (DHLs) were obtained from the Agronomy Department, Faculty of Agriculture, Al-Azhar University, Nasr City, Cairo, Egypt, and from published literature [37]. The GD between the 16 wheat genotypes was determined using SSR molecular markers.

### DNA extraction and SSR analysis

DNA was isolated by sampling 0.5 g of fresh leaves from each genotype and crushing in liquid nitrogen. Genomic DNA of each genotype was extracted using the Wizard Genomic DNA Purification Kit (PROMEGA Corporation Biotechnology, Madison, Wisconsin, USA). DNA concentration was measured spectrophotometrically (Thermo Scientific, USA) at 260 nm, and the extract was electrophoresed on 0.8% agarose to check the quality. The purified DNA was standardized at 25 ng μl^-1^ as final concentration and stored at -20°C. To provide coverage of the entire wheat genome, sixty SSR markers were used in this study based on several previous studies [38–43] and the Grain Genes database (http://wheat.pw.usda.gov/ggpages/maps.shtml); 16 of the 60 markers were specific SSR markers linked to earliness or yield and yield components in wheat (S2 Table). PCRs were performed in 20 μl reaction volume containing 10 μl of 1× GoTaq green master mix (Promega Corporation, Madison, USA), 0.5 μl primer, 1.5 μl of 25 ng genomic DNA, and 7.5 μl nuclease-free water. The thermal cycler profile of PCR for the SSR analysis included initial denaturation at 94°C for 3 min, followed by 40 cycles of denaturation at 94°C for 1 min, annealing at 51–61°C (depending on the individual SSR primers) for 1 min, an extension at 72°C for 2 min, followed by final extension at 72°C for 10 min. The PCR products were separated using capillary electrophoresis by using a QI Axcel Advanced system device (Qiagen, Hilden, Germany).

### Field trial

**Fig 1:**
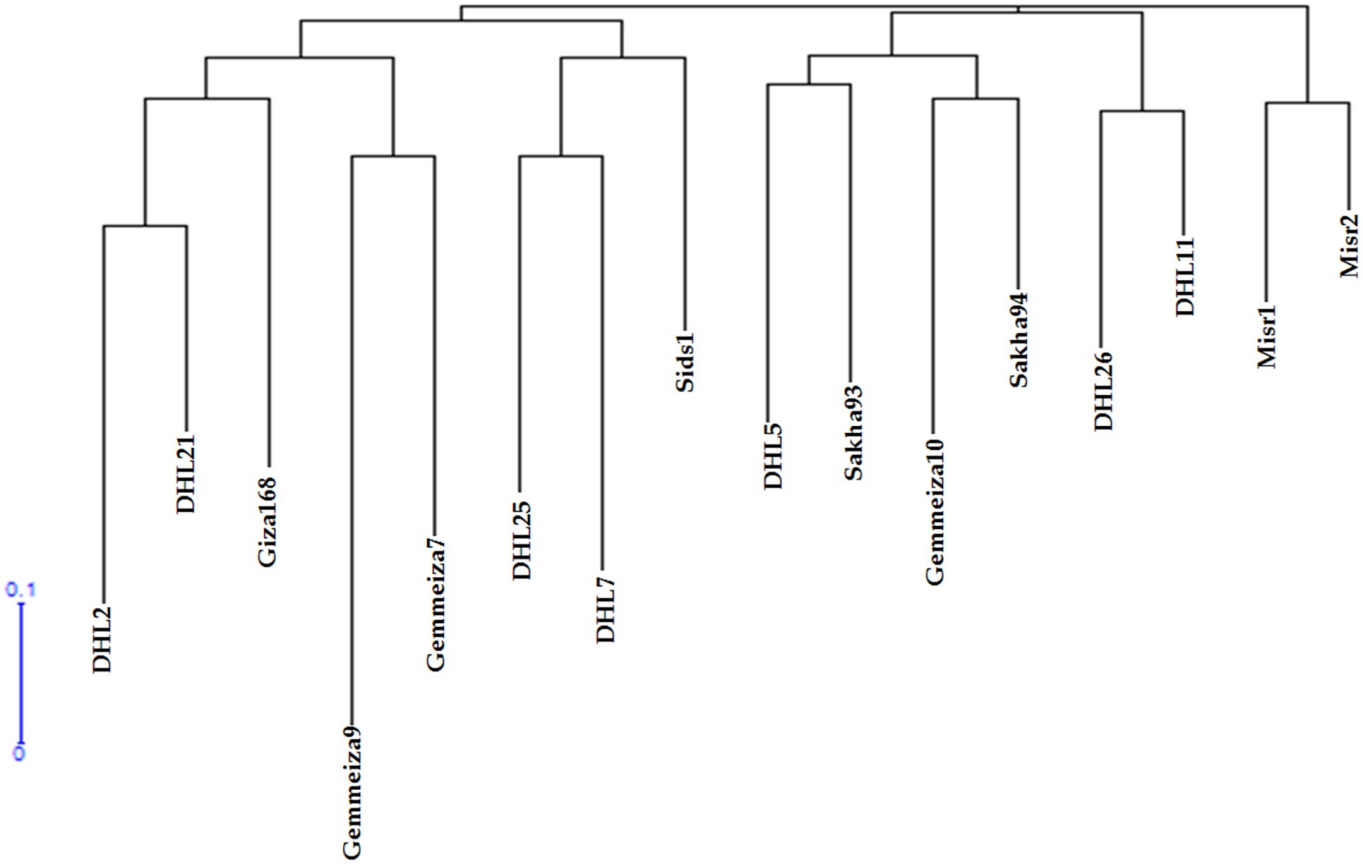
Dendrogram obtained using NJ clustering procedures in 16 wheat genotypes by using SSR data based on Jaccard genetic dissimilarity index. DHL: Doubled haploid line.

The experiment was performed at the King Saud University Agricultural Research Station (24°42ʹN, 44°46ʹE, 400 m asl). In the first season, the half-diallel mating system was used, which includes n parents and one set of F1s excluding reciprocals by using n (n—1)/2 crosses. The eight parental genotypes, i.e., five varieties—Gemmeiza-7, Giza-168, Gemmeiza-9, Sakha-93, and Misr1—and three DHLs—DHL21, DHL7, and DHL2—were crossed; they were selected owing to their greater genetic diversity based on the findings of SSR markers (Table 1 and Fig 1), to produce a total of 28 F_1_ crosses. The eight parental genotypes and their 28 F_1_ crosses were investigated in a randomized complete block design with three replications. The planting date was December 15 in 2017 and December 20 in 2018 at a seedling rate of 360 germinating kernels m^-2^, which constitutes the heat stress condition, with an average temperature of 30.4–31.0/14.2–14.4 °C day/night during grain filling duration in the two seasons (after optimum planting date one month). The experimental unit (plot) consisted of six rows (3.0 m long) each, with the distance between rows of 0.17 m.

**Table 1:** Genetic Distance (GD) estimation between the 16 wheat genotypes by using SSR molecular markers.

GD	Sakha93	DHL5	Sakha94	Gemmeiza10	DHL11	DHL7	Sids1	Misr2	DHL26	DHL21	DHL2	DHL25	Misr1	Giza168	Gemmeiza7
DHL5	0.484														
Sakha94	0.444	0.469													
Gemmeiza10	0.563	0.500	0.400												
DHL11	0.394	0.516	0.382	0.393											
DHL7	0.911	0.652	0.750	0.750	0.630										
Sids1	0.556	0.594	0.364	0.581	0.355	0.714									
Misr2	0.432	0.455	0.235	0.485	0.324	0.688	0.353								
DHL26	0.469	0.552	0.583	0.633	0.286	0.565	0.531	0.441							
DHL21	0.515	0.633	0.485	0.667	0.576	0.667	0.516	0.424	0.567						
DHL2	0.816	0.690	0.657	0.806	0.706	0.739	0.735	0.676	0.621	0.440					
DHL25	0.600	0.654	0.625	0.692	0.586	0.556	0.400	0.606	0.630	0.615	0.778				
Misr1	0.556	0.636	0.412	0.581	0.406	0.580	0.485	0.250	0.484	0.467	0.735	0.621			
Giza168	0.510	0.656	0.595	0.727	0.559	0.754	0.629	0.541	0.500	0.581	0.506	0.690	0.450		
Gemmeiza7	0.794	0.692	0.613	0.625	0.571	0.500	0.607	0.594	0.667	0.600	0.740	0.682	0.500	0.692	
Gemmeiza9	0.883	0.857	0.794	0.760	0.733	0.805	0.767	0.735	0.786	0.821	0.846	0.875	0.724	0.704	0.700

### Trait measurements and data collection

The plants from the middle rows (guarded) were used to reduce environmental impact for both the parents and F_1_ crosses to measure the following earliness traits (days to heading, days to maturity, and grain filling duration). Days to heading (DH, days) was recorded as the number of days from sowing to the date when 50% of spikes had emerged from the flag leaf. Days to maturity (DM, days) was recorded as the number of days from sowing to the date when 50% of spikes on the top of the peduncles had turned yellow. Grain filling duration (GFD, days) was the number of days from anthesis to maturity. Plant height (PH, cm plant^-1^) was measured from the topsoil to the tip of the spikes excluding awns at the time of maturity. After harvesting, the agronomic traits, including grain yield (GY, ton ha^-1^), harvest index (HI, %), number of spikes (NS, m^-2^), spike length (SL, cm), number of spikelets (NSS, spike^-1^), number of kernels (NKS, spike^-1^), hundred kernel weight (HKW, g), and grain filling rate (GFR, %), were determined. GFR is the rate at which assimilates are transferred from the source to the sink; it was calculated as the ratio between GY and GFD.

### Statistical analysis

Genotyping Data Analysis: SSR data were scored visually for allele size and presence or absence for each primer. SSR bands were scored as qualitative characters, e.g., present (1) or absent (0), to create a binary matrix. A matrix to evaluate pairwise genetic dissimilarity between genotypes was calculated on the basis of the Jaccard dissimilarity coefficient; hierarchical neighbor joining (NJ) method was performed using the DARWIN 6.0.021 software program [44]. The principal coordinate analysis (PCoA) was performed using the Jaccard dissimilarity coefficient matrix to reduce the dimensions of data space by using the XLSTAT statistical package (Version 2018; Excel Add-ins soft SARL, New York, NY, USA).

Phenotyping Data Analysis: The data for all the studied traits were subjected to analysis of variance (ANOVA) by using SAS software (Version 9.2; SAS Institute, Inc., Cary, North Carolina, USA). The sum of squares (SS) of parents vs hybrids error was calculated as follows: SS of parents vs that of hybrids _(Residual error (P. vs. H))_ = SS pooled error _(total)_—SS of parents error _(Error p)_—SS of hybrids error _(Error H)_, as described by Utz [45]. The combined analysis was performed across the two seasons according to Gomez and Gomez [46], after test the homogeneity of error variance. Heterotic effects were computed relative to mid-parent heterosis (MPH) and better-parent heterosis (BPH) as follows: MPH % = (F_1_ value—parent mean)/parent mean × 100; BPH % = (F_**1**_ value—better parent)/better parent × 100, where F_1_ is the hybrid performance, and better parent was the one with better traits. MPH and BPH were calculated to test the significance of the heterotic effects according to the formula suggested by Wynne et al. [47]. Combining ability analysis was performed according to method 2, model 1 [48]. Heritability (broad sense and narrow sense) and genetic gain were calculated, as described by Burton and Devane [49] and Al-Ashkar et al. [50]. Pearson’s correlation coefficient matrix analysis between all studied parameters and GD and heterosis, and stepwise multiple linear regression (SMLR) analysis were performed using XLSTAT statistical package (Version 2018; Excel Add-ins soft SARL, New York, NY, USA). Path analysis was used to partition correlation coefficients into direct and indirect effects by using a previously described method [51], with GY (y) considered as a response variable and DM (x_1_), GFD (x_2_), HKW (x_3_), and NKS (x_4_) considered as explanatory variables. The residual value was calculated using the following equation:

$$1=\text{Residual}\;\text{value}+\text{direct}\;\text{effect}({\text{x}}_{1}^{2}+{\text{x}}_{2}^{2}+{\text{x}}_{3}^{2}+{\text{x}}_{4}^{2})\text{on}(\text{y})+\text{indirect}\;\text{effect}[(2\;{\text{x}}_{1}{\text{r}}_{12}{\text{x}}_{2})+(2\;{\text{x}}_{1}{\text{r}}_{13}{\text{x}}_{3})+(2\;{\text{x}}_{1}{\text{r}}_{14}{\text{x}}_{4})+(2\;{\text{x}}_{2}{\text{r}}_{23}{\text{x}}_{3})+(2{\text{x}}_{2}{\text{r}}_{24}{\text{x}}_{4})+(2\;{\text{x}}_{3}{\text{r}}_{34}{\text{x}}_{4})]\;\text{on}(\text{y}).$$

## Results and analysis

### Genetic relationships and diversity patterns

A genetic dissimilarity matrix based on the Jaccard coefficient was generated using the microsatellite data. This matrix was used to group all accessions by using NJ. The estimated Jaccard coefficient among genotypes varied from 0.235 to 0.911, with an average of 0.597. The highest genetic dissimilarity (0.911) was noted between Sakha93 and DHL7, whereas the lowest (0.235) was between Sakha94 and Misr2 (Table 1). These relationships were consistent with the results of hierarchical cluster analysis (Fig 1). The 16 genotypes were divided into three major groups with clear separation from each other as follows: the first group (I) included two genotypes (Misr2 and Misr1). The second group divide into two sub-clusters, the first (II) with two genotypes (DHL11 and DHL26) and the second (III) with four genotypes (DHL5, Sakha93, Sakha94 and Gemmeiza10). The third group divide into two sub-clusters, the first (V) with three genotypes (DHL7, DHL25 and Sids1) and the second divided into two (VI and IV) groups (Gemmeiza7 and Gemmeiza9) and (DHL21 DHL2 and Giza 168), respectively (Fig 1).

**Fig 2:**
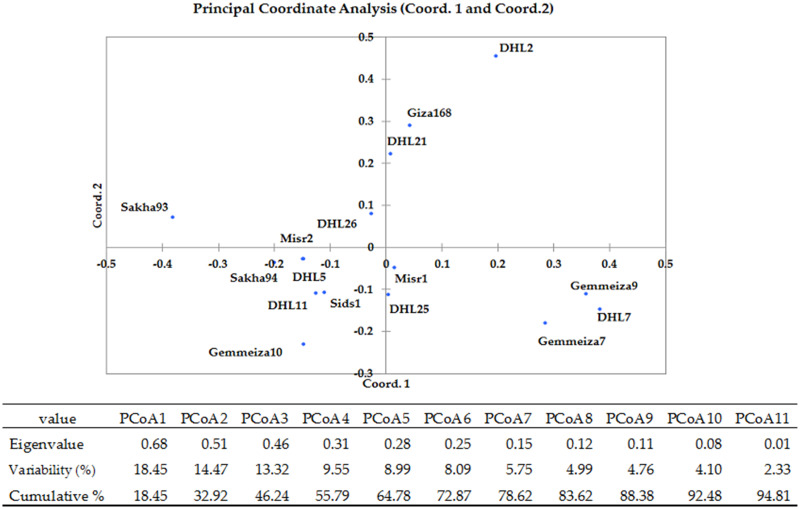
Principal coordinates analysis (PCoA) among the 16 wheat genotypes performed using SSR data based on Jaccard genetic dissimilarity index. DHL: Doubled haploid line.

Two-dimensional PCoA revealed wide genetic dissimilarity between most of the genotypes, explaining 94.81% of the total variation (Fig 2). All genotypes were distributed into four quadrants (groups). Quadrants 1 and 2 included three and five genotypes, respectively. Quadrant 4 contained only two genotypes. Quadrant 3 was the largest and included six genotypes; it covered less PCoA area than quadrant 2 (Fig 2). The genotypes (two-dimensional PCoA) were distributed with an acceptable level, and the results were consistent with those of hierarchical clustering analysis.

### Earliness parameters and yield performance of parents and F_1_ hybrids

Phenotyping data analysis (Table 2) revealed that the investigated genotypes (parents and hybrids) had highly significant mean squares for all studied traits in each and across the two seasons. The earliness parameters and yield performance of the parents and their F_1_ hybrids are shown in S3 Table. In S1, S2, and combined, the DH of the F_1_ generation ranged from 74.23 to 92.56 days, 69.36 to 88.79 days, and 71.80 to 90.68 days, with an average of 81.86, 77.86, and 79.86 days, respectively, for 28 hybrid combinations. Among all hybrid combinations for the combined data, five were significantly earlier in heading than the parent. Hybrid (P2 × P7) showed heading after 72.30 days, which was 9.47% below the average, and hybrid (P6 × P8) showed heading after of 90.68 days, which was 13.54% above the average (S3 Table).

**Table 2:** Analysis of variance for days to heading, grain filling duration, and grain yield for each and across seasons of parents and their F_1_ hybrids in bread wheat genotypes.

S.O.V.	d.f.		Days to heading			Grain filling duration			Grain yield		
	S	Comb	S1	S2	Comb	S1	S2	Comb	S1	S2	Comb
Replications	2	4	5.21	10.23	7.72	1.59	2.54	2.06	0.002	0.56	0.26
Season (S)		1			1034.1**			462.07**			23.31**
Genotypes (G)	35	35	91.74**	82.76**	127.15**	19.62**	18.31**	26.44**	9.20**	7.28**	13.42**
Parents (P)	7	7	113.13**	101.92**	135.76**	13.11**	12.49**	25.55**	7.03**	8.94**	10.33**
Error (P)	14	14	8.62	6.95	15.56	2.91	2.87	6.08	0.193	0.301	0.50
Hybrids (H)	27	27	85.43**	77.09**	129.64**	19.10**	17.74**	25.67**	9.71**	7.04**	14.15**
Error (H)	54	54	6.97	6.65	13.61	3.10	3.24	6.28	0.208	0.284	0.45
P *vs*.H	1	1	112.18**	101.54**	213.59**	79.17**	74.40**	153.53**	10.33**	2.12**	16.07**
Residual error (P *vs*.H)	2	2	0.47	2.14	2.61	0.64	4.01	4.65	0.55	0.011	0.06
G × S		35			34.49**			8.62**			4.16**
P × S		7			72.86**			1.47			5.65**
H × S		27			25.48*			10.17**			3.89**
P *vs*.H × S		1			9.13			17.04			0.91
Pooled error	70	140	7.11	6.58	6.84	3.00	3.20	3.10	0.201	0.281	0.22

* = significant at *p* ≤ 0.05,

** = significant at *p* ≤ 0.01.

In S1, S2, and combined, the GFD for the F_1_ generation ranged from 49.90 to 58.56 days, 42.59 to 52.72 days, and 44.91 to 55.31 days, with an average of 54.41, 49.36, and 51.86 days, respectively, for 28 hybrid combinations. Among all hybrid combinations for the combined data, six had significantly longer filling period than the corresponding parent, ranging from 47.99 to 52.38 days. Regarding GY, the yield performance of the hybrids in S1, S2, and combined ranged from 3.97 to 11.33 ton ha^-1^, 3.37 to 10.29 ton ha^-1^, and 3.67 to 10.71 ton ha^-1^, with an average of 8.68, 7.64, and 8.17 ton ha^-1^, respectively, for 28 hybrid combinations. In combined data, four hybrids showed 20% greater yield than the average value (8.17 ton ha^-1^), and four hybrids showed 10–20% greater yield than the average. Hybrids (P2 × P7) had GY of 10.71 ton ha^-1^, which was 31.08% above the average, and hybrid (P1 × P2) had GY of 3.67 ton ha^-1^, which was 55.12% below the average (S3 Table).

### Heterosis of mid-parent and high-parent for earliness traits and yield performance of the F_1_ generation

The ANOVA showed that mean squares as an indication of heterosis over all crosses were highly significant between parents and hybrids in both the seasons (Table 2). The interaction between parents *vs* hybrids and seasons was insignificant, indicating that the response of heterosis was similar in magnitude in both the seasons. Therefore, the heterosis estimates were calculated on the basis of combined data for both the seasons. For DH, the MPH ranged from -13.39 to 11.71, and BPH ranged from -10.46 to 20.54 (Table 3). Five of the 28 hybrids showed MPH of more than 10%, and only one showed BPH of more than 10%. For GFD, the MPH ranged from -11.68 to 12.74, and BPH ranged from -14.26 to 12.17. Of the 28 hybrid combinations, only three showed MPH of more than 10%, and only two showed BPH of more than 10%. For GY, the MPH ranged from -36.39 to 46.22. In addition, eight hybrids showed MPH of more than 20%, representing 28% of the hybrid combinations; five hybrids showed MPH of 10 to 20%, representing 18% of the hybrid combinations. For GY, the BPH ranged from -40.83 to 37.73. Six hybrids showed BPH of more than 20%, representing 21% of the hybrid combinations; three hybrids showed BPH of 10 to 20%, representing 10% of the hybrid combinations. Five hybrids exhibited MPH and BPH exceeding 20% in yield (themselves). Hybrids (P2 × P4) and (P2 × P5) showed the best MPH and BPH for DH (-11.57% and -7.65%; -13.39% and -8.36%, respectively), GFD (12.74% and 12.17%; 12.09% and 10.59%, respectively), and GY (36.04% and 20.04%; 44.06% and 37.73%, respectively; Table 3).

**Table 3:** Mid-parent heterosis (upper right) and better-parent heterosis (lower left) in F_1_ hybrids for days to heading, grain filling duration, and grain yield for combined data across seasons.

Hybrids	Traits	Giza168	Sakha93	DHL21	Gemmeiza9	DHL7	Misr1	DHL2	Gemmeiza7
Giza168	DH		0.84	-6.13	-9.85	-11.80	-6.40	2.05	2.81
	GFD		4.69	5.31	9.49	8.55	0.98	-7.73	4.75
	GY		-36.39	-4.12	16.07	46.22	-16.75	-12.13	23.81
Sakha93	DH	5.72		-0.42	-11.57	-13.39	-10.55	-12.40	2.30
	GFD	2.30		-1.83	12.74	12.09	10.75	8.48	0.15
	GY	-40.83		-28.14	36.04	44.06	14.52	37.07	-16.04
DHL21	DH	-1.79	-0.22		-4.24	-7.09	-3.01	-3.15	4.58
	GFD	4.12	-5.13		5.25	7.34	1.13	3.41	4.50
	GY	-22.70	-38.48		2.99	12.40	3.79	-9.32	10.54
Gemmeiza9	DH	-1.11	-7.65	0.21		-2.37	-3.26	-8.28	7.60
	GFD	7.52	12.17	2.22		3.50	4.72	-0.03	4.01
	GY	-3.80	20.04	6.79		-8.72	20.52	16.44	-12.33
DHL7	DH	-2.48	-8.86	-2.03	-1.65		-5.82	-1.49	5.33
	GFD	7.49	10.59	5.12	2.63		3.90	2.04	7.09
	GY	30.47	37.73	28.19	-16.10		33.94	8.91	6.16
Misr1	DH	2.17	-7.02	1.02	-2.83	-4.70		2.18	11.71
	GFD	-1.53	5.59	-0.28	0.33	0.36		-1.69	-11.68
	GY	-31.19	0.74	7.22	20.09	22.71		0.08	7.68
DHL2	DH	9.47	-10.46	-0.80	-6.34	1.35	3.87		1.00
	GFD	-10.70	2.67	1.20	-4.92	-2.17	-2.44		5.08
	GY	-31.22	13.59	-5.54	8.28	-6.30	-6.63		24.18
Gemmeiza7	DH	3.93	6.06	8.20	16.66	15.11	20.54	7.11	
	GFD	4.27	-1.69	2.86	2.61	6.54	-14.26	1.25	
	GY	4.43	-24.50	17.25	-14.19	-0.45	5.03	13.20	
L.S.D.		DH			GFD			GY	
		MPH	BPH		MPH	BPH		MPH	BPH
0.05		1.60	1.84		2.13	2.46		1.11	1.28
0.01		2.11	2.43		2.81	3.25		1.46	1.69

Doubled haploid line (DHL), days to heading (DH, day), grain filling duration (GFD, day), and grain yield (GY, ton ha^-1^).

### Genetic parameters and combining ability performance

The SCA of hybrids and the general combining ability (GCA) of parents were highly significant for the three traits studied (DH, GFD, and GY) in S1, S2, and combined. The GCA/SCA ratios for the three traits were more than 1 in S1, S2, and combined (Table 4). Heritability (narrow sense and broad sense) and genetic gain showed DH of 51.65%, 81.24%, and 13.49%, respectively, GFD of 51.03%, 73.28%, and 11.11%, respectively, and GY of 46.41%, 69.47%, and 25.63%, respectively (Table 4). We selected eight hybrids with yield heterosis of 20% above the average for evaluating the combining ability effects (Table 5). Regarding DH, the mean values varied from 71.80 to 84.65 days; MPH, from -13.39 to 2.81; GCA, from -5.06 to 2.68; and SCA, from -6.26 to 0.04. Regarding GFD, the mean values of hybrids ranged from 51.90 to 54.62 days; MPH from 3.90 to 12.74; GCA, from -0.03 to 1.08; and SCA, from -0.34 to 3.71. Concerning GY, the mean values of hybrids showed significant variations from 8.11 to 10.71 ton ha^-1^; MPH from 20.52 to 46.22; GCA, from -1.52 to 1.11; and SCA (for the eight robust heterosis combinations), from 0.73 to 2.49. In accordance with the findings of cluster analysis, among the strong heterosis combinations, the cross types were group I with groups V, and VI; group III with groups V, VI and IV; group V with group VI; and group VI with group IV (Table 5). Therefore, groups I and III were thought to produce some strong heterosis combinations.

**Table 4:** Mean squares of general (GCA) and specific (SCA) combining ability for days to heading, grain filling duration, and grain yield for each and across seasons of parents and their F_1_ hybrids in bread wheat genotypes, and heritability and genetic gain values for traits across seasons.

S.O.V.	d.f.		Days to heading			Grain filling duration			Grain yield			
	S	Comb	S1	S2	Comb	S1	S2	Comb	S1	S2	Comb
GCA	7	7	75.88**	68.44**	72.11**	19.03**	31.37**	25.20**	6.12**	7.21**	6.62**
SCA	28	28	19.25**	17.37**	18.30**	4.88**	7.26**	6.07**	2.30**	1.23**	1.68**
GCA × S		7			52.20**			17.93**			7.71**
SCA × S		28			11.33**			5.37**			1.96**
Error	70	140	2.37	2.19	2.28	0.71	1.07	0.89	0.07	0.09	0.07
GCA/SCA			3.94	3.90	3.92	3.89	4.32	4.15	2.66	5.86	2.94
GCA × S/GCA					0.73			0.71			0.93
SCA × S/SCA					0.62			0.77			1.40
Heritability: narrow sense					51.65			51.03			46.41
Heritability: broad sense					81.24			73.28			69.47
Genetic gain					13.49			11.11			25.63

* = significant at *p* ≤ 0.05,

** = significant at *p* ≤ 0.01.

**Table 5:** Relationship between group division and strong combination yield, mid-parent heterosis, GCA and SCA.

Hybrids	Mean performance			Mid-parent heterosis			SCA			GCA			SSR Clustering Combination Type
	DH	GFD	GY	DH	GFD	GY	DH	GFD	GY	DH	GFD	GY	
P1 × P5	71.80	53.51	8.87	-11.80	8.55	46.22	-5.03	2.33	1.71	-5.06,2.11	-0.16, 0.32	-1.52, 0.12	IV × V
P1 × P8	76.52	51.90	8.11	2.81	4.75	23.81	0.23	1.63	1.26	-5.06,1.08	-0.16, -1.08	-1.52, 0.08	IV × VI
P2 × P4	74.57	53.83	9.73	-11.57	12.74	36.04	-4.61	3.12	1.62	-2.75,1.66	-.0.03, -0.34	-0.83, 0.42	III × VI
P2 × P5	73.59	53.98	9.36	-13.39	12.09	44.06	-6.04	3.61	2.49	-2.75,2.11	-0.03, 0.32	-0.83, 0.12	III × V
P2 × P7	72.30	54.62	10.71	-12.40	8.48	37.07	-6.26	3.71	1.49	-2.75,1.05	-0.03, 0.86	-0.83, 1.11	III × IV
P4 × P6	84.65	52.56	9.81	-3.26	4.72	20.52	0.04	1.31	0.73	1.66,2.68	-0.34, 0.07	0.42, 0.43	VI × I
P5 × P6	83.02	52.57	10.02	-5.82	3.90	33.94	-2.05	-0.34	1.46	2.11,2.68	0.32, 0.07	0.12, 0.43	V × I
P7 × P8	80.58	53.87	10.68	1.00	5.08	24.18	-1.32	2.07	1.53	1.05,1.08	0.86, -1.08	1.11, 0.08	IV × VI

Special combining ability (SCA), general combining ability (GCA), days to heading (DH, day), grain filling duration (GFD, day), and grain yield (GY, ton ha^-1^). Giza168 (P1), Sakha93 (P2), DHL21 (P3), Gemmeiza9 (P4), DHL7 (P5), Misr1 (P6), DHL2 (P7), and Gemmeiza7 (P8)

### Correlation coefficients between GD of parents and heterosis and SCA effects

Correlation analysis between GD and each heterosis (MPH and BPH) and SCA effects based on SSRs is shown in Table 6. GD showed significant and positive association (*p* < 0.05) with GFD (r = 0.487, 475, and 0.359), NKS (r = 0.385 and 0.375), HKW (r = 0.389 and 0.378), HI (r = 0.670, 679, and 0.531), GY (r = 0.406, 0.379, and 0.395), and GFR (r = 0.444, 0.502, and 0.424), respectively. Furthermore, GD was negatively and significantly correlated (*p* < 0.05) with heterosis (MPH and BPH) and SCA for DH (r = -0.420, 0.406, and -0.418). The DM, PH, NS, SL, and NSS traits showed insignificant correlations between GD and each heterosis (MPH and BPH) and SCA effects (Table 6).

**Table 6:** Pearson correlation coefficient of GD with MPH, BPH, and SCA of yield and yield-related traits for combined data across seasons.

Trait	Parameters	Correlation	Trait	Parameters	Correlation	Trait	Parameters	Correlation
DH	MPH	-0.420*	NS	MPH	0.103	HKW	MPH	0.241
	BPH	-0.406*		BPH	0.096		BPH	0.389*
	SCA	-0.418*		SCA	0.065		SCA	0.378*
DM	MPH	-0.175	SL	MPH	-0.027	HI	MPH	0.670**
	BPH	-0.297		BPH	-0.113		BPH	0.679**
	SCA	-0.220		SCA	-0.097		SCA	0.531**
GFD	MPH	0.487**	NSS	MPH	0.281	GY	MPH	0.406*
	BPH	0.475**		BPH	0.263		BPH	0.379*
	SCA	0.359*		SCA	0.104		SCA	0.395*
PH	MPH	-0.150	NKS	MPH	0.295	GFR	MPH	0.444*
	BPH	-0.355		BPH	0.385*		BPH	0.502**
	SCA	-0.136		SCA	0.375*		SCA	0.424*

Mid-parent heterosis (MPH), better-parent heterosis (BPH), special combining ability (SCA), days to heading (DH, days), days to maturity (DM, days), grain filling duration (GFD, days), plant height (PH, cm), number of spikes (NS, m^2^), spike length (SL, cm), number of spikelets per spike (NSS), number of kernels per spike (NKS), hundred kernel weight (HKW, g), harvest index (HI, %), grain yield (GY, ton ha^-1^), and grain filling rate (GFR, %).

### Identification of traits related to yield performance

**Fig 3:**
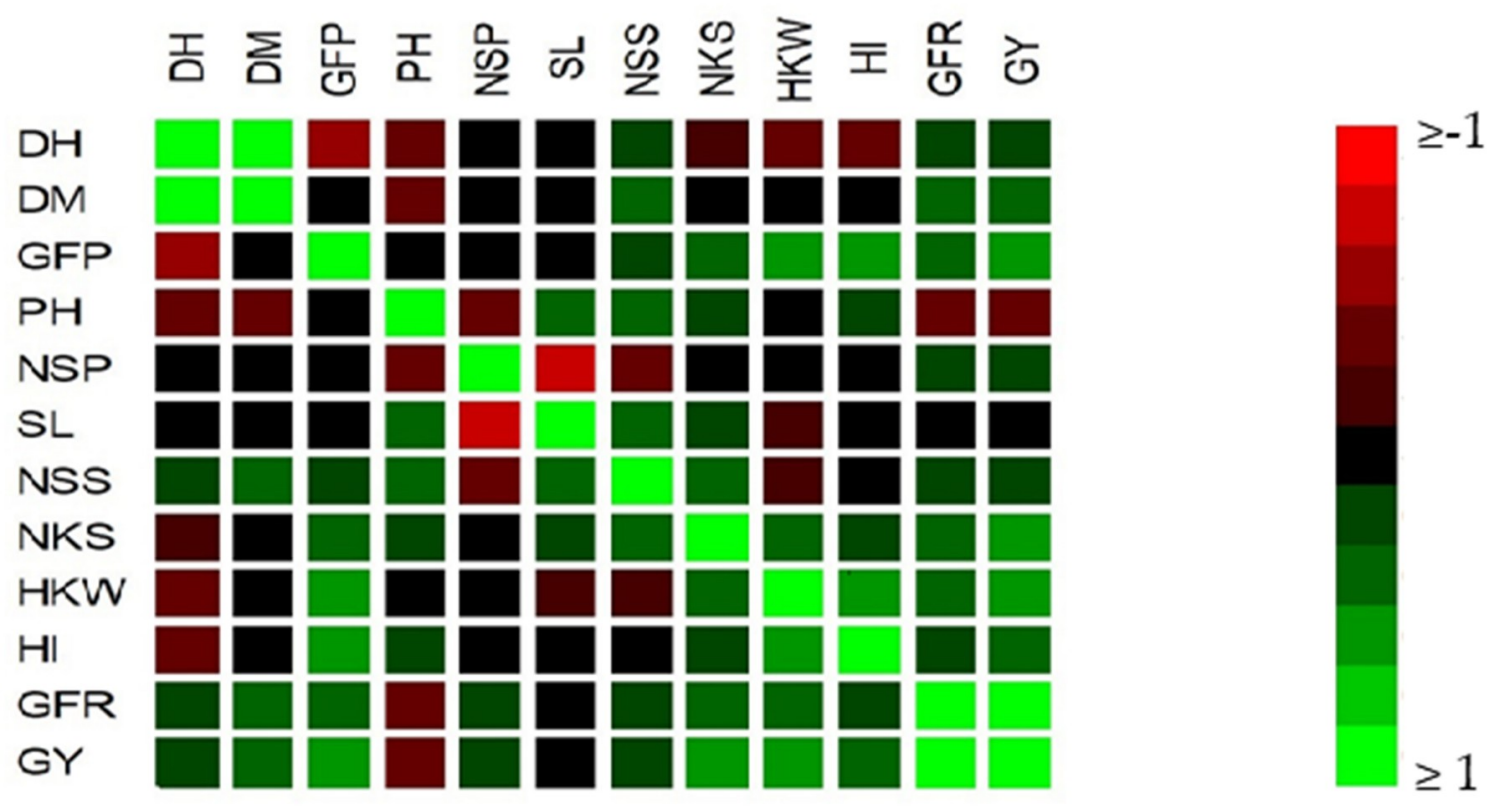
Correlation matrix between the 12 measured traits of wheat genotypes for combined data (n = 34). Days to heading (DH, days), days to maturity (DM, days), grain filling duration (GFD, days), plant height (PH, cm), number of spikes (NS, m^2^), spike length (SL, cm), number of spikelets per spike (NSS), number of kernels per spike (NKS), hundred kernel weight (HKW, g), harvest index (HI, %), grain yield (GY, ton ha^-1^), and grain filling rate (GFR, %).

The yield-related traits and the extent of their contribution to yield were determined by performing correlation analysis between all traits (Fig 3). Correlation analysis of F_1_ generation and their respective parents showed significant and positive association for five measured parameters with GY (*p* < 0.05, r = 0.43–0.98). GFD showed significant and positive association with four measured parameters (*p* < 0.05, r = 0.42–0.60). DH showed insignificant correlations with all measured parameters excluding DM and GFD (*p* < 0.05, r = 0.87 and -0.48). The correlation analysis results indicated that DM, GFD, NKS, HKW, and GFR are significant parameters, considering their positive contribution to GY (Table 7). The results of SMLR showed that GFD, DM, HKW, and NKS were significantly correlated to GY, and their contribution rates were 0.294, 0.241, 0.111, and 0.076, respectively (Table 7); after the GFR parameter was removed, the R^2^ of this model was 0.722. The path coefficient analysis partitioned the SMLR values into direct and indirect effects via alternate characters or pathways (Table 7). The traits considered very important by SMLR (GFD, HKW, DM, and NKS) were applied in the correlation and path analyses. The components of GY variation were determined directly and jointly by each factor. Each direct and indirect effect contributed to 0.498 and 0.224, respectively. The R^2^ value was 0.722, with noise value of 0.527. Thus, GFD, HKW, DM, and NKS traits could be considered as important criteria for the selection of hybrids for yield. Days to maturity (DM), grain filling duration (GFD), number of kernels per spike (NKS), hundred kernel weight (HKW), coefficient partial determination (R^2^ Par.), cumulative coefficient determination (R^2^ Com.), * means *P* value of coefficient partial determination.

**Table 7:** Stepwise regression and path coefficient analyses for grain yield (dependent variable) with four yield-related traits (independent variables) for combined data across seasons.

Source	Stepwise regression				Path coefficient			
					Partitioning the correlation			R^2^
	Regression Coefficient	*P* value*	R^2^ Par.	R^2^ Com.	Direct effect	Indirect effect	Correlation value	Direct effect
Intercept	-142.954	<0.0001						
GFD	0.692	0.010	0.294	0.294	0.256	0.229	0.484	0.065
DM	0.673	0.001	0.241	0.535	0.487	-0.058	0.429	0.237
HKW	4.772	0.040	0.111	0.646	0.321	0.160	0.481	0.103
NGS	0.294	0.043	0.076	0.722	0.304	0.178	0.482	0.093
Indirect effect								0.224
Total R^2^				0.722				0.722
Residual				0.527				0.527

## Discussion

Genotyping and molecular marker development as well as recombinant DNA technology provide the potential to achieve advanced knowledge and to create valuable tools to assist in the selection and breeding of novel plant varieties with enhanced photosynthetic efficiency, increased biotic stress tolerance, or better performance under unfavorable abiotic conditions. This, in turn, allows the creation of a new generation of sustainable crops [52]. Genetic identification of wheat cultivars by using morphological analyses is usually inaccurate, especially in the early stages of plant development [50]. Furthermore, morphological analyses frequently require a large set of phenotypic data and more cropping seasons for screening and evaluation, which are often hindered by high environmental influences. The heterogeneity in agricultural soil adversely impacts field evaluation, resulting in high coefficient of variation, which affects the reliability of the results [53]. The advantages of genetic fingerprinting techniques are that the DNA content of a cell is not affected by environmental conditions, growth stage, and organ type [54]. Moreover, they seem to be a promising tool for predicting heterosis in many species, for example, rice [55], maize [56], rape [57], and wheat [58].

Hybrid crop breeding mainly involves the determination of parents having high genetic diversity [59] that can be crossed to produce F_1_ hybrids with robust heterosis and allow the identification of new genes (alleles) from the rich existing allelic stock for some landraces and cultivated wheat varieties in order to generate hybrid vigor [60, 61]. Previous studies have shown that genetic dissimilarity for 26 microsatellite loci ranged from 0.43 to 0.94, with an average distance of 0.77, for 998 cultivars of bread wheat [14]. In our study, the GD varied from 0.235 to 0.911, with an average distance of 0.597 (Table 1). This is in well agreement with the findings of a study suggesting that the average polymorphism information content across ten elite Iranian bread wheat lines was 0.503 by using SSR markers [13]. In this study, the SSR marker analysis performed using numerous primers was effective in distinguishing wheat genotypes, which indicates the increased efficiency of using these markers for GD analysis of these genotypes. Jaccard genetic dissimilarity index revealed clusters that consisted of three major groups. Some Sub-groups included large number genotypes, suggesting high similarity in their morphological traits (Fig 1).

The eight parental genotypes selected in this study showed large genetic diversity among themselves, indicating increased potential for strong out-crossing and higher performance of F_1_ hybrid varieties, which are essential for the occurrence of heterosis [27–29, 62]. Furthermore, AHC and PCoA revealed compatible relationships among these genotypes. The results of phenotyping data analysis were consistent with those of genotyping data analysis: the mean squares for genotypes, parents, and crosses were highly significant for all studied traits, indicating the existence of sufficient genetic variability among the genotypes for the studied traits (Table 2). The mean squares for all traits were significant in the two seasons, with mean values in S1 being higher than those in S2 for three traits, which can be attributed to the increased temperature during the grain filling period in S2. The mean squares for genotypes, parents, and crosses were significant for the three studied traits across the two seasons, indicating that the performance of the genotypes differed from one season to another.

Since crossing is mainly performed to exploit heterosis, negative values of heterosis for earliness traits (e.g., DH and DM), but positive values for yield traits (e.g., NSP, NKS, HKW, and GY) are desired. Plant breeders need to break the negative correlation between DH and GY by extending the GFD. Thus, the complexity of inheritance for measured trait properties in wheat genotypes becomes evident. In most cases, the beneficial effect of a given from parents on progeny in relation to one of the traits was not correlated with an enhancement of the other trait and often leads to even further decrease. That is, the occurrence of heterosis for one trait cannot necessarily be equivalent to that of another trait [10]. In the same hybrid, some traits might show positive heterosis, whereas others may show negative heterosis [63].

The GD between parents can be used to produce hybrids with high yield and earliness. We found significant and positive or negative MPH and BPH for the examined traits. Furthermore, two crosses [(P2 × P4) and (P2 × P5)] showed the highest performance in DH, GFD, and GY, and both had large genetic diversity among themselves (0.911 and 0.883, respectively); therefore, they were deemed as promising hybrids (Table 1, S3, and 4). Few studies have estimated GD before producing F_1_ hybrids for improving the crossing effectiveness to generate heterosis in wheat. Nevertheless, the association between GD and heterosis remains unclear. Significant relationship between GD and heterosis has been shown for water absorption, dough development, grain weight, and grain-yield traits in wheat [3, 10, 64]. Our results showed that GD was positively and significantly correlated with heterosis effects for GFD, HI, GY, and GFR, indicating the potential of molecular markers for predicting hybrid performance [62]. In contrast, GD was negatively and significantly correlated with DH, indicating its dominance for earliness (Table 6). These traits (DM, PH, NS, SL and NASS) showed a weak relationship between GD and heterosis. In contrast, Legesse et al. [65] reported positive and significant correlation between PH and GD. Melchinger et al. [66], Betrán et al. [67] and Wegary et al. [62] stated that the level of correlations between GD, and heterosis depend on the genotype used. Several reasons have been proposed for the low relationship of GD with heterosis such as absence of a linkage between genes controlling the traits measured, uneven genome coverage, varied impact of dominance and random marker distribution [62, 68]. Expectation of heterosis using molecular markers would be useful when a high proportion (50%) of the markers used to calculate of GD are linked with QTL affecting heterosis of the target trait in the genotype used in the study [69]. The variation in the relationship between GD and hybrid performance can be attributed to the difference in genetic materials used in the studies and the significant effect of environmental factors on the relative amount of heterosis [50, 55, 70].

The ANOVA showed significant variances for both GCA and SCA (*p* > 0.01). The variances of SCA were lower than those of GCA for DH, GFD, and GY in S1, S2, and combined, indicating that additive genes primarily controlled the inheritance of these traits. Previous studies on wheat [10, 71] also revealed large differences in variances of GCA than of SCA for GY. Heritability information, quantitative effects of genetic and environmental variation (broad sense), and ratio of the additive genetic variance to phenotypic variance (narrow sense) [50], can be used to predict the credibility of the phenotypic value, which is indicative of the breeding value. However, heritability information alone is not sufficient for the prediction without the involvement genetic gain. After narrow-sense heritability and genetic gain were checked and considered, the performance of the hybrids for DH, GFD, and GY was found to be moderate (Table 5). This indicated that additive and non-additive genetic variance was very closely related with the three studied traits, and they cannot be effectively relied upon during the selection process [72]. In our study, in which eight hybrids with yield heterosis of 20% above the average were chosen, showed highly significant and positive SCA in seven hybrids, including two types of combinations of good × good and good × poor general combiners. However, parents having high GCA might not necessarily yield high SCA. For instance, the hybrids (P4 × P6), (P5 × P6), and (P7 × P8) had parents with a good general combiner. In contrast, the hybrids (P1 × P5), (P1 × P8), (P2 × P4), (P2 × P5), and (P2 × P7), which involved one good and one poor general combiner for GY, yielded high SCA, indicating high genetic diversity of the parents (Table 5).

In some cases, hybrids had significant and positive as well as high SCA effects even when their parents were both negative and poor general combiners; this can be attributed to the genetic diversity between the parents. Moreover, crossing of parents having low GCA effects produced relatively high magnitude of non-additive gene effects, resulting in high SCA effects [73, 74]. Interestingly, two hybrids (P2 × P4) and (P2 × P5) were considered as promising, as they showed highly useful heterosis, high SCA effects, and involved at least one parent as a good general combiner. The results of correlation analysis between the GD of parents and SCA effects was consistent with those obtained using heterosis (Table 6). Many researchers have reported significant correlations between GD and yield-related traits and the suitability of molecular distance for predicting single-cross performance [3, 10, 62, 64]. According to Benin et al. [64], the lack of correlation can come back to additive gene effects for the traits and/or parents involved in the crosses have the same genes, making the expression of SCA unexpected and at random. However, if markers correlated with specific traits are chosen, their use in assessing genetic diversity and hence hybrid performance can be more competent [75]. Therefore, comprehension of hybrid performance and SCA and the detection of various heterosis groups is important for hybrid wheat breeding programs.

Correlation and multivariate analyses (stepwise regression and path coefficient) are important tools for understanding the relationship between yield and its related traits [76]. The traits chosen based on simple correlation coefficients without the consideration of interactions among yield and related traits may not be useful for establishing fundamental breeding programs [77]. Our results showed that GY was positively and significantly related with DM, GFD, NS, NKS, HKW, HI, and GFR; these correlations themselves reflect only the degree of trait interrelations (Fig 3). The ineffective impact of the used traits on yield in the regression model was removed by performing stepwise regression after excluding the GFR trait. In this study, GFD, HKW, DM, and NKS, in the order of importance, were found to be reliable traits for GY (*p* < 0.01, Table 6). The stepwise regression model had a coefficient of determination (R^2^) of 0.722. Many investigators have used this model (e.g., [78–80]). Based on the results of correlation and stepwise regression, we used path analysis to classify the four chosen traits according to their direct and indirect effects. If the correlation between two traits was owing to a direct effect, indicating a relationship between them, they were selected for performance improvement [80]. The partitioning of the correlation coefficients into direct and indirect effects was close for GFD; however, for HKW, NKS, and DM, the direct effect was greater than the indirect effect (Table 7). The partitioning of the coefficient of determination for direct and indirect effects was 0.498 and 0.224, respectively, and most of the indirect effect was attributed to GFD. Hence, we concluded that HKW, MD, and NKS are good traits for predicting GY.

In conclusion, our results confirmed the importance of SSR markers as an effective tool for evaluating wheat GD, which varied from 0.235 to 0.911 between the 16 genotypes examined in this study. In general, the distance measure could be helpful for the detection of genetically similar/different genotypes. The eight parental genotypes selected in this study showed large genetic diversity among themselves, indicating increased potential for strong out-crossing and higher performance of F_1_ hybrid varieties, which are essential for the occurrence of heterosis. Thus, we considered the two crosses (P2 × P5) and (P2 × P4) that showed the highest performance in DH, GFD, and GY and had large genetic diversity among themselves (0.911 and 0.883, respectively) as promising hybrids of heat stress tolerance.

## Supporting information

S1 Table Names and pedigree of the 16 bread wheat cultivars and Doubled Haploid Lines (DHLs) used in this study. (DOCX)

S2 Table Microsatellite (SSR) markers used in this study and location on the chromosome. Markers in bold are specific SSR markers linked to earliness or yield and yield components in wheat.
(DOCX)

S3 Table Mean performance of 28 F_1_ hybrid combinations and their respective parents for days to heading, grain filling duration, and grain yield for each and across seasons. (DOCX)
