# Supplementary material for: Selection criteria for high-yielding and early-flowering bread wheat hybrids under heat stress
Source: PLoS One. 2020 Aug 12;15(8):e0236351. doi: 10.1371/journal.pone.0236351 (PMC7423122; doi:10.1371/journal.pone.0236351)
Supplement: S1 Table — (DOCX) [file pone.0236351.s001.docx]

**Table S1.** Names and pedigree of the 16 bread wheat cultivars and doubled haploid lines (DHLs) used in this study.

| **Name** | **Pedigree** | |
| --- | --- | --- |
| Giza-168 | | MRL/BUC//SERICM 93046-8 M-OY-OM-2Y-OB-OGZ. |
| Gemmeiza-7 | | CMH74A.630/SX//SERI82/AGENTCGM 4611-2GM-3GM-1GM-OGM |
| Gemmeiza-9 | | Ald“s”/Huac//CMH74 .630/SxCGM 4583 -5GM- 1GM- OGM |
| Sakha-93 | | Sakha 92/TR810328 S8871-IS-2S-IS-0S |
| Sakha94 | | Opata/Rayon//Kauz |
| Gemmeiza10 | | Maya74"S"/on//1160-147/3/BB/GLL14/chat"S"/5/Crow"S"/5/ Crow"s"  CGM5820-3GM-1GM-2GM-0GM |
| Sids1 | | HD2172/2/Pavon//1158.57/Maya74SD46 -45D-15D-05D |
| Misr1 | | OASSIS / SKAUZ//4*BCN/3/2*PATOR CMSS00Y01881T-050M-030Y-030M-030WGY-33M-0Y-0S |
| Misr2 | | SKAUZ/BAV92 CMSS96M03611S- IM-010SY-010M-010SY-8M-0Y-0S |
| DHLs (21,25) | | Derived from the cross (Line-115 × Gemmeiza-7) (El-Hennawy et al. 2011) |
| DHLs (5,7,11) | | Derived from the cross (Line-115 × Giza-164) (El-Hennawy et al. 2011) |
| DHL26 | | Derived from the cross (Gemmeiza-7× Giza-164) (El-Hennawy et al. 2011) |
| DHL2 | | Derived from the cross (Giza-164× Giza-168) (El-Hennawy et al. 2011) |
