# Supplementary material for: Selection criteria for high-yielding and early-flowering bread wheat hybrids under heat stress
Source: PLoS One. 2020 Aug 12;15(8):e0236351. doi: 10.1371/journal.pone.0236351 (PMC7423122; doi:10.1371/journal.pone.0236351)
Supplement: S2 Table — Markers in bold are specific SSR markers linked to earliness or yield and yield components in wheat. (DOCX) [file pone.0236351.s002.docx]

**Table S2.** Microsatellite (SSR) markers used in this study and location on the chromosome. Markers in bold are specific SSR markers linked to earliness or yield and yield components in wheat

| **Chromosome** | **Markers on chromosome** | **Chromosome** | **Markers on chromosome** |
| --- | --- | --- | --- |
| 1A | Xgwm136**,** Xwmc9 | 4B | Xgwm251, Xwmc310 |
| 1B | Xbarc188, [**Xbarc80**](https://wheat.pw.usda.gov/cgi-bin/GG3/report.cgi?class=marker;query=*barc80*;name=Xbarc80)**,** Xgwm413, Xgwm18 | 4D | Xwmc206, Xcfd84 |
| 1D | [**Xbarc62**](https://wheat.pw.usda.gov/cgi-bin/GG3/report.cgi?class=locus;name=Xbarc62)**,** [**Xcfd63**](https://wheat.pw.usda.gov/cgi-bin/GG3/report.cgi?class=marker;query=*cfd63*;name=Xcfd63)**, Xgdm111,** Xgwm155, Xgwm458 | 5A | [**Xwmc489**](https://wheat.pw.usda.gov/cgi-bin/GG3/report.cgi?class=locus;name=Xwmc489-5A)**,** [**Xgwm617**](https://wheat.pw.usda.gov/cgi-bin/GG3/report.cgi?class=marker;query=*gwm617*;name=Xgwm617) |
|  |  | 5B | Xgwm335, Xcfd60 |
| 2A | [**Xgwm448**](https://wheat.pw.usda.gov/cgi-bin/GG3/report.cgi?class=locus;name=Xgwm448)**,** [**Xgwm122**](https://wheat.pw.usda.gov/cgi-bin/GG3/report.cgi?class=locus;name=Xgwm122), Xgwm312, Xgwm249, Xgwm210 | 5D | Xgwm174, Xcfd18, Xcfd19 |
|  |  | 6A | Xcfd1, Xwmc145 |
| 2B | Xgwm614, Xwmc175 | 6B | **Xgwm132,** Xcfd13 |
| 2D | Xgwm539, Xgwm455 | 6D | [**Xcfd188**](https://wheat.pw.usda.gov/cgi-bin/GG3/report.cgi?class=locus;name=Xcfd188)**,** [**Xbarc196**](https://wheat.pw.usda.gov/cgi-bin/GG3/report.cgi?class=locus;name=Xbarc196)**,** Xcfd49 |
| 3A | Xbarc12, Xwmc11, [**Xbarc67**](https://wheat.pw.usda.gov/cgi-bin/GG3/report.cgi?class=locus;name=Xbarc67), [**Xwmc664**](https://wheat.pw.usda.gov/cgi-bin/GG3/report.cgi?class=marker;query=*wmc664*;name=Xwmc664), [**Xbarc310**](https://wheat.pw.usda.gov/cgi-bin/GG3/report.cgi?class=marker;query=*barc310*;name=Xbarc310), [**Xbarc356**](https://wheat.pw.usda.gov/cgi-bin/GG3/report.cgi?class=marker;query=*barc356*;name=Xbarc356) | 7A | Xwmc17, Xwmc225 |
|  |  | 7B | [**Xgwm577**](https://wheat.pw.usda.gov/cgi-bin/GG3/report.cgi?class=marker;query=*gwm577*;name=Xgwm577)**,** Xbarc182 |
| 3B | Xgwm340, Xgwm299, Xgwm389 | 7D | Xgwm295, Xcfd46Xcfd46, Xcfd66, Xgwm350, |
| 3D | Xgwm314, Xgwm3 |  |  |
| 4A | Xgwm160, Xgwm350 |  |  |
