# Supplementary material for: Selection criteria for high-yielding and early-flowering bread wheat hybrids under heat stress
Source: PLoS One. 2020 Aug 12;15(8):e0236351. doi: 10.1371/journal.pone.0236351 (PMC7423122; doi:10.1371/journal.pone.0236351)
Supplement: S3 Table — (DOCX) [file pone.0236351.s003.docx]

Table S3. Mean performance of 28 F_1_ hybrid combinations and their respective parents for days to heading, grain filling duration, and grain yield for each and across seasons.

| Genotypes | | | Days to heading (DH, day) | | |  | Grain filling duration (GFD, day) | | |  | Grain yield (GY, ton ha^-1^) | | |
| --- | --- | --- | --- | --- | --- | --- | --- | --- | --- | --- | --- | --- | --- |
|  |  |  | S1 | S2 | Comb |  | S1 | S2 | Comb |  | S1 | S2 | Comb |
| Giza168 | | (P1) | 74.56 | 72.68 | 73.62 |  | 52.23 | 47.32 | 49.78 |  | 5.47 | 5.20 | 5.33 |
| Sakha93 | | (P2) | 82.90 | 78.59 | 80.75 |  | 49.90 | 45.11 | 47.51 |  | 6.25 | 6.15 | 6.20 |
| DHL21 | | (P3) | 82.56 | 78.29 | 80.43 |  | 53.23 | 48.62 | 50.93 |  | 9.19 | 8.23 | 8.71 |
| Gemmeiza9 | | (P4) | 90.23 | 85.55 | 87.89 |  | 50.23 | 45.75 | 47.99 |  | 8.79 | 7.43 | 8.11 |
| DHL7 | | (P5) | 91.56 | 86.81 | 89.19 |  | 51.23 | 46.39 | 48.81 |  | 7.10 | 6.50 | 6.80 |
| Misr1 | | (P6) | 88.90 | 85.32 | 87.11 |  | 54.90 | 49.86 | 52.38 |  | 8.74 | 7.59 | 8.17 |
| DHL2 | | (P7) | 86.56 | 82.09 | 84.33 |  | 55.56 | 50.84 | 53.20 |  | 9.86 | 9.01 | 9.43 |
| Gemmeiza7 | | (P8) | 77.23 | 73.22 | 75.23 |  | 51.23 | 47.41 | 49.32 |  | 8.32 | 7.21 | 7.76 |
| P1 × P2 | | | 79.90 | 75.76 | 77.83 |  | 53.23 | 48.61 | 50.92 |  | 3.97 | 3.37 | 3.67 |
| P1 × P3 | | | 74.23 | 70.37 | 72.30 |  | 55.56 | 50.49 | 53.03 |  | 7.25 | 6.21 | 6.73 |
| P1 × P4 | | | 74.23 | 71.37 | 72.80 |  | 55.90 | 51.14 | 53.52 |  | 8.24 | 7.36 | 7.80 |
| P1 × P5 | | | 74.23 | 69.36 | 71.80 |  | 56.56 | 50.45 | 53.51 |  | 9.72 | 8.01 | 8.87 |
| P1 × P6 | | | 77.23 | 73.21 | 75.22 |  | 53.90 | 49.26 | 51.58 |  | 5.99 | 5.25 | 5.62 |
| P1 × P7 | | | 82.23 | 78.95 | 80.59 |  | 49.90 | 45.12 | 47.51 |  | 7.00 | 5.97 | 6.49 |
| P1 × P8 | | | 78.56 | 74.47 | 76.52 |  | 54.23 | 49.57 | 51.90 |  | 8.72 | 7.50 | 8.11 |
| P2 × P3 | | | 82.90 | 77.59 | 80.25 |  | 50.56 | 46.07 | 48.32 |  | 5.72 | 4.99 | 5.36 |
| P2 × P4 | | | 76.56 | 72.57 | 74.57 |  | 56.90 | 50.76 | 53.83 |  | 10.33 | 9.13 | 9.73 |
| P2 × P5 | | | 75.56 | 71.62 | 73.59 |  | 57.90 | 50.06 | 53.98 |  | 10.00 | 8.72 | 9.36 |
| P2 × P6 | | | 76.56 | 73.59 | 75.08 |  | 57.90 | 52.72 | 55.31 |  | 8.80 | 7.66 | 8.23 |
| P2 × P7 | | | 74.23 | 70.37 | 72.30 |  | 58.56 | 50.68 | 54.62 |  | 11.14 | 10.29 | 10.71 |
| P2 × P8 | | | 81.90 | 77.66 | 79.78 |  | 50.90 | 46.07 | 48.49 |  | 6.35 | 5.37 | 5.86 |
| P3 × P4 | | | 82.23 | 78.95 | 80.59 |  | 54.56 | 49.55 | 52.06 |  | 9.25 | 8.07 | 8.66 |
| P3 × P5 | | | 80.90 | 76.69 | 78.80 |  | 55.90 | 51.16 | 53.53 |  | 9.19 | 8.23 | 8.71 |
| P3 × P6 | | | 82.90 | 79.59 | 81.25 |  | 54.23 | 50.24 | 52.24 |  | 9.35 | 8.16 | 8.76 |
| P3 × P7 | | | 81.90 | 77.66 | 79.78 |  | 56.23 | 51.45 | 53.84 |  | 8.69 | 7.76 | 8.22 |
| P3 × P8 | | | 83.56 | 79.22 | 81.39 |  | 54.90 | 49.86 | 52.38 |  | 9.71 | 8.50 | 9.10 |
| P4 × P5 | | | 88.23 | 84.65 | 86.44 |  | 52.56 | 47.63 | 50.10 |  | 7.33 | 6.28 | 6.80 |
| P4 × P6 | | | 86.90 | 82.39 | 84.65 |  | 54.90 | 50.21 | 52.56 |  | 10.04 | 9.58 | 9.81 |
| P4 × P7 | | | 80.56 | 77.39 | 78.98 |  | 53.56 | 47.60 | 50.58 |  | 10.85 | 9.57 | 10.21 |
| P4 × P8 | | | 89.56 | 85.95 | 87.76 |  | 52.90 | 48.31 | 50.61 |  | 7.38 | 6.54 | 6.96 |
| P5 × P6 | | | 85.23 | 80.8 | 83.02 |  | 53.90 | 51.24 | 52.57 |  | 10.54 | 9.50 | 10.02 |
| P5 × P7 | | | 87.23 | 83.7 | 85.47 |  | 54.56 | 49.53 | 52.05 |  | 9.43 | 8.24 | 8.84 |
| P5 × P8 | | | 88.90 | 84.28 | 86.59 |  | 54.90 | 50.19 | 52.55 |  | 8.17 | 7.28 | 7.73 |
| P6 × P7 | | | 89.90 | 85.27 | 87.59 |  | 54.23 | 49.57 | 51.90 |  | 9.28 | 8.33 | 8.81 |
| P6 × P8 | | | 92.56 | 88.79 | 90.68 |  | 47.23 | 42.59 | 44.91 |  | 9.16 | 7.99 | 8.58 |
| P7× P8 | | | 83.23 | 77.92 | 80.58 |  | 55.90 | 51.83 | 53.87 |  | 11.33 | 10.02 | 10.68 |
| Parents | L.S.D. 0.05 | | 4.78 | 4.29 | 5.52 |  | 2.78 | 2.90 | 3.45 |  | 0.72 | 0.90 | 0.99 |
|  | L.S.D. 0.01 | | 6.35 | 5.70 | 5.95 |  | 3.69 | 3.85 | 3.72 |  | 0.95 | 1.20 | 1.07 |
| Hybrids | L.S.D. 0.05 | | 4.30 | 4.20 | 4.21 |  | 2.87 | 2.90 | 2.86 |  | 0.74 | 0.87 | 0.76 |
|  | L.S.D. 0.01 | | 5.71 | 5.57 | 5.56 |  | 3.81 | 3.85 | 3.78 |  | 0.99 | 1.15 | 1.01 |
